# The active immune role of keratinocytes: from passive targets to key hubs in psoriatic inflammation

**DOI:** 10.3389/fimmu.2026.1970701

**Published:** 2026-09-29

**Authors:** Yu Pei

**Affiliations:** Department of Dermatology, The Second Affiliated Hospital of Harbin Medical University, Harbin, Heilongjiang, China

**Keywords:** epidermal barrier, epithelial-immune reciprocity, IL-23/Th17 axis, IL-36, keratinocytes, metabolic reprogramming, psoriasis

## Abstract

Psoriasis is a chronic immune-mediated inflammatory skin disease historically viewed as a disorder of keratinocyte hyperplasia, with subsequent recognition of central immune cell involvement. However, accumulating multi-omics and functional genomic evidence has reshaped this paradigm, positioning keratinocytes not merely as passive targets of immune attack but as active participants within a bidirectional epithelial-immune network that drives inflammatory initiation, maintenance, and amplification. This critical narrative review synthesizes the molecular mechanisms by which keratinocytes integrate genetic susceptibility, environmental triggers, metabolic reprogramming, and cell death programs to modulate psoriatic inflammation. We examine interleukin (IL)-1/IL-36 cytokines, the dual function of the antimicrobial peptide LL-37, danger signal release and dendritic cell recruitment, chemokine networks and exosome-mediated communication, the neuropeptide-keratinocyte axis, carbohydrate and lipid metabolic abnormalities, mitochondrial dysfunction and reactive oxygen species (ROS)-mediated feedback, epidermal barrier defects and microbiome dysregulation, diagnostic biomarkers, and therapeutic innovations. We further critically evaluate controversies, conceptual challenges, research gaps, and future directions. A keratinocyte-centric framework—situated within the broader context of epithelial-immune reciprocity—advances understanding of psoriasis pathogenesis and informs precision therapies targeting the barrier-immune axis.

## Introduction

Psoriasis is a chronic immune-mediated inflammatory skin disease characterized by epidermal hyperproliferation, abnormal differentiation, and excessive immune cell infiltration in the dermis and epidermis (1, 2). Historically, psoriasis was initially viewed primarily as a disorder of epidermal keratinocyte hyperproliferation. Subsequent advances established the central contribution of T-cell-mediated immune responses, particularly the IL-23/Th17 axis, leading to a predominantly immune-centric model of disease pathogenesis. More recent multi-omics and functional studies have further refined this framework by highlighting bidirectional interactions between keratinocytes and immune cells. Modern studies confirm that psoriasis involves a complex interaction network among keratinocytes, dendritic cells (DCs), T lymphocytes, neutrophils, macrophages, natural killer (NK) cells, and mast cells (1, 3, 4). Within this network, keratinocytes are no longer merely passive targets of immune attack or simple effectors; rather, they function as active immune regulators that directly participate in the initiation, maintenance, and amplification of inflammation through the secretion of cytokines, chemokines, and antimicrobial peptides (5–7). This paradigm shift from a single immune axis to a multidimensional epithelial-immune interaction network not only reshapes understanding of psoriasis etiology but also provides a theoretical basis for developing novel precision therapies targeting keratinocytes and their microenvironment (2, 8).

The traditional immunological model of psoriasis emphasizes the central role of the IL-23/Th17 axis, positing that activated DCs produce IL-23 to drive Th17 cell differentiation, which in turn secretes IL-17A and other factors acting on keratinocytes to cause hyperproliferation (4, 9). Although this model successfully guided the development of biologics, accumulating evidence indicates that keratinocytes play an earlier and more active role in the disease cascade. Single-cell RNA sequencing data show that keratinocytes and DCs in human psoriatic lesions both express IL23A, and in animal models keratinocyte-derived IL-23 is closely associated with IL-36 expression, suggesting that keratinocytes themselves are producers of key pro-inflammatory factors rather than merely responders (10). Furthermore, keratinocytes can upregulate major histocompatibility complex class II (MHC-II) molecules to present self-antigens such as keratin 17 (K17), specifically activating CD4+ T cells, or directly promote T cell activation by expressing co-stimulatory molecules such as inducible T-cell co-stimulator ligand (ICOSLG), demonstrating non-professional antigen-presenting cell function (11, 12). Keratinocytes also recruit and activate macrophages through leucine-rich alpha-2-glycoprotein 1 (LRG1)-enriched extracellular vesicles (EVs), or regulate Th17 cell skin recruitment through sphingosine-1-phosphate receptor 2 (S1PR2) signaling, demonstrating bidirectional active regulation of both innate and adaptive immunity (13, 14). These findings support a central, active role for keratinocytes within bidirectional immune interactions and show that self-amplifying feedback loops with immune cells contribute to psoriasis chronicity (5, 15).

As the first line of the skin barrier, keratinocyte immune activity is an important component of the initiation phase of psoriatic inflammation. Under environmental triggers such as infection and stress, keratinocytes rapidly release danger signals and innate immune mediators. For example, IL-1/IL-36 family signaling is particularly important in pustular psoriasis, with keratinocytes serving as major local sources and responders within this inflammatory circuit (16–22); receptor-interacting protein kinase 3 (RIPK3) in keratinocytes not only mediates necroptosis but also directly activates the IL-36alpha/nuclear factor kappa B (NF-kappaB) pathway through non-necrotic mechanisms, further amplifying inflammatory signaling (23). In addition, keratinocyte-derived LL-37 functions as both a self-antigen and an immune adjuvant, bridging innate and adaptive immunity (24–26). At the cell death level, keratinocyte pyroptosis mediated by gasdermin D (GSDMD) or gasdermin E (GSDME) not only causes barrier disruption but also releases large amounts of pro-inflammatory contents such as IL-1beta and IL-18, directly inducing or exacerbating dermatitis (27–29). Metabolic reprogramming is also an important basis for inflammation initiation: metabolic enzymes such as pyruvate kinase M2 (PKM2) and phosphoglycerate mutase 1 (PGAM1) are highly expressed in keratinocytes, driving cell proliferation through aerobic glycolysis and enhancing the activity of inflammatory signaling pathways such as NF-kappaB (30–32). These mechanisms collectively indicate that keratinocytes, by integrating genetic susceptibility, environmental stimuli, metabolic status, and cell death programs, constitute a key hub for psoriasis inflammation initiation, and their dysfunction may occur early in the inflammatory cascade and can contribute to subsequent immune-cell recruitment.

This critical narrative review synthesizes the scientific evidence for the transition of keratinocytes from passive targets to active immune hubs in psoriasis, focusing on molecular mechanisms in inflammation initiation, maintenance, and metabolic reprogramming. We examine transcriptomic and epigenetic remodeling in psoriasis-like keratinocytes (33), lncRNA UCA1-mediated inflammatory regulation (34), and cell-specific alterations in ERAP1/ERAP2 expression (35); analyze multidimensional interaction networks between keratinocytes and T cells, DCs, neutrophils, and sensory neurons (3, 36); and summarize novel diagnostic biomarkers and therapeutic targets based on keratinocyte activity. Literature was selected from PubMed and Web of Science with emphasis on publications from 2018 to 2026, supplemented by earlier landmark studies where necessary, prioritizing original research and authoritative reviews in psoriasis immunology; inclusion was based on direct relevance to keratinocyte immune function in psoriasis. References were cross-checked for bibliographic accuracy and relevance to the cited statements. In terms of clinical translation, although current biologics targeting IL-17/IL-23 are highly effective, some patients experience primary or secondary failure, and epidermal barrier function cannot be fully restored (37). Targeting keratinocyte-relevant pathways (e.g., TYK2, IL-36, pyroptosis, and metabolic programs) represents an important direction for next-generation therapeutic strategies (29, 38, 39). For example, topical TYK2 inhibition can directly suppress keratinocyte pro-inflammatory signaling while limiting systemic exposure (38); targeting GSDMD or NLRP3 inflammasome activity may interrupt inflammation-barrier feedback (29, 40); and modulating the bile acid-farnesoid X receptor (FXR) axis or carbohydrate and lipid metabolism may correct abnormal keratinocyte phenotypes (39, 41). By integrating these advances, this review aims to provide theoretical support and practical pathways for precise phenotyping, biomarker development, and barrier-immune synergistic repair strategies in psoriasis (**Figure 1**).

**Figure 1:**
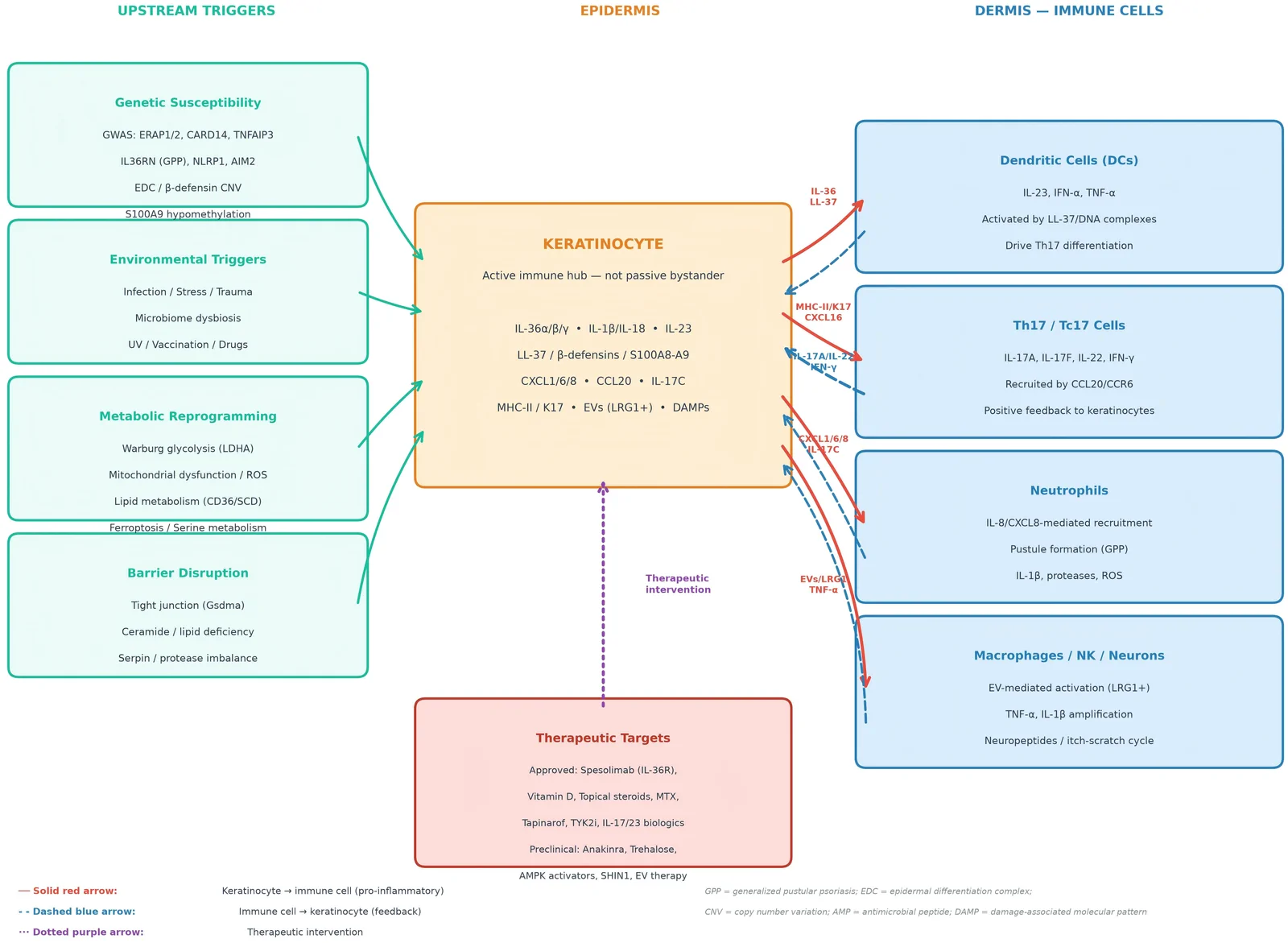
Keratinocytes as active hubs in psoriatic inflammation: epithelial-immune reciprocity. Genetic and epigenetic susceptibility, environmental triggers, metabolic reprogramming, and barrier disruption converge on keratinocytes, which function as active immune hubs rather than passive targets. Keratinocytes release IL-36, IL-1, IL-23, LL-37, chemokines, DAMPs, and EVs that interact with dendritic cells, Th17/Tc17 cells, neutrophils, macrophages, NK cells, and sensory neurons; immune-cell-derived IL-17A, IL-22, and IFN-gamma provide feedback that sustains keratinocyte activation. Therapeutic strategies shown span different evidence levels, from approved therapies to off-label/limited clinical use and preclinical or investigational approaches; inclusion does not imply equivalent clinical validation. GPP, generalized pustular psoriasis; EDC, epidermal differentiation complex; CNV, copy number variation; AMP, antimicrobial peptide; DAMP, damage-associated molecular pattern. (Figure generated using matplotlib).

## Genetic susceptibility and environmental triggers: molecular basis of keratinocyte active responses

### Psoriasis risk genes: specific expression and function in keratinocytes

Recent genome-wide association studies (GWAS) and multi-omics investigations have identified more than 70 genome-wide significant psoriasis loci and continue to expand the catalogue; some risk genes are expressed or functionally active in keratinocytes and may shape epidermal immune responses (42–44). It is important to distinguish several categories of evidence that are often conflated: (i) common polygenic susceptibility loci identified through GWAS; (ii) rare monogenic or Mendelian disorders that phenocopy psoriasis; (iii) differential gene expression in lesional keratinocytes; (iv) functional evidence from keratinocyte-specific gene manipulation in animal models; (v) causal-variant colocalization studies; and (vi) genes identified primarily through bioinformatic prioritization. These evidence classes differ substantially in their mechanistic implications and should not be treated as equivalent.

Among keratinocyte-intrinsic inflammatory programs, absent in melanoma 2 (AIM2) is significantly upregulated in lesional keratinocytes, where it not only releases IL-1beta and IL-18 through inflammasome-dependent pathways to mediate inflammation but also regulates keratinocyte programmed cell death and proliferation in an inflammasome-independent manner, and may participate in trained immunity mechanisms during relapse (45). Loss of negative regulatory factors is equally critical: Sprouty1 expression is reduced in psoriatic skin and keratinocytes, and restoring its expression inhibits Stat3 activation and cathelicidin (LL-37 precursor) production, thereby reducing DC and IL-17A+ gammadeltaT cell infiltration, confirming that failure of keratinocyte-intrinsic inhibitory pathways underlies excessive innate immune activation (46). At the epidermal differentiation complex (EDC) on chromosome 1q21, multiple psoriasis susceptibility loci encode barrier and antimicrobial proteins, including late cornified envelope genes and S100 family members; copy number variation of beta-defensin genes at the DEFB locus has been associated with increased psoriasis risk (47), and S100A9 is hypomethylated and overexpressed in psoriatic lesions (48). As an antimicrobial peptide released from keratinocytes under threat and a damage-associated molecular pattern (DAMP), S100A9 can activate TLR-dependent innate immune signaling and cytokine expression. Evidence from primary keratinocyte models supports the involvement of TLR4-associated inflammatory pathways, while immune-cell-derived effects also contribute to the broader S100A9-mediated inflammatory network (49, 50). The epigenetic regulation of S100A9 suggests a likely involvement in psoriasis etiology.

Rare monogenic disorders provide mechanistic insights distinct from common polygenic susceptibility. For example, rare loss-of-function mutations in ADAR1 cause keratinocytes and melanocytes to become major sources of type I interferon, upregulating interferon-stimulated gene expression through reduced RNA editing, an effect reversible by JAK/TYK2 inhibitors (51). Although biologically informative, this represents a monogenic interferonopathy rather than common polygenic psoriasis susceptibility. Similarly, keratinocyte-specific deletion of A20 in mice spontaneously induces psoriasis-like lesions dependent on the MyD88 pathway rather than congenital interferon receptors, demonstrating that impaired NF-kappaB negative feedback in epidermal cells is sufficient to initiate a lymphocyte-mediated inflammatory cascade (52); this is functional mechanistic evidence from an animal model, not human population-genetic susceptibility evidence. Transcriptomic and bioinformatic analyses further reveal that multiple targets including IL2RA, COMP, and A2ML1 are aberrantly expressed in keratinocytes, and that genes such as ASF1A and RARRES2 harbor shared causal variants (53, 54). These distinct evidence categories collectively shape the molecular phenotype of keratinocytes as inflammation-initiation hubs, but their relative contributions to human disease pathogenesis differ and warrant explicit distinction (see **Table 1** for a stratified summary of psoriasis susceptibility genes and risk loci with keratinocyte-relevant functions).

**Table 1:** Selected psoriasis susceptibility genes and risk loci with keratinocyte-relevant functions, stratified by evidence class.

Gene/locus	Evidence class	Keratinocyte-relevant function	Ref.
ERAP1/ERAP2	GWAS susceptibility	Antigen processing; MHC-I peptide loading in keratinocytes	(42, 43)
AIM2	Expression + functional	Inflammasome activation; IL-1beta/IL-18 release; keratinocyte death/proliferation	(45)
IL36RN	Monogenic (GPP)	IL-36Ra deficiency; uncontrolled IL-36 signaling in pustular psoriasis	(17, 18)
ADAR1	Monogenic (rare)	RNA editing defect; type I interferon production by keratinocytes	(51)
TNFAIP3 (A20)	Functional (mouse KO)	NF-kappaB negative feedback; keratinocyte-specific deletion causes psoriasis-like lesions	(52)
DEFB (beta-defensin cluster)	Copy number variation	Antimicrobial peptide production; innate immune defense in epidermis	(47)
S100A9	Epigenetic + expression	Hypomethylation/overexpression; DAMP; TLR-dependent inflammatory signaling, including TLR4-associated pathways	(48–50)
LCE3C_LCE3B-del	GWAS (EDC)	Barrier protein deletion; epidermal differentiation complex	(42)
CARD14	GWAS + monogenic	NF-kappaB activation in keratinocytes; familial psoriasis	(42, 44)
IL23A	Functional (scRNA-seq)	Keratinocyte-derived IL-23; links innate and adaptive immunity	(10)
Sprouty1 (SPRY1)	Functional + expression	Negative regulator of Stat3; reduced in psoriatic keratinocytes	(46)
ASF1A, RARRES2	Colocalization + bioinformatics	Shared causal variants; keratinocyte expression abnormalities	(53, 54)

### Environmental factors inducing keratinocyte phenotypic transition

Environmental triggers interact with the host genetic background to induce epigenetic remodeling and transcriptional reprogramming in keratinocytes, shifting them from homeostatic maintenance to a pro-inflammatory phenotype. Microbial stimulation is an important environmental trigger: *in vitro* experiments show that Staphylococcus epidermidis treatment significantly alters the keratinocyte transcriptome, and differentially expressed genes overlap with skin microbiome GWAS candidate genes, suggesting that commensal-host genetic interaction directly affects epidermal immune activity (55). Inflammatory stress itself is a core driver of phenotypic transition: under conditions mimicking mild and severe psoriasis-like inflammation, keratinocytes exhibit 858 and 6,987 differentially expressed genes, respectively, and the transcriptomic profile of mildly inflamed cells more closely resembles clinical psoriatic epidermis, driven primarily by IL-17 pathway genes (33). This phenotypic transition is accompanied by profound epigenetic modifications: inflammation causes dysregulation of histone acetyltransferases, deacetylases, and DNA methyltransferases, with a global trend toward decreased DNA methylation and specific alterations in 5-hydroxymethylcytosine patterns (33). Non-coding RNAs also participate in amplification of environmental signals: IL-17A induces keratinocyte overexpression of miR-378a, which enhances NF-kappaB signaling by targeting and inhibiting NFKBIA/IkappaBzeta, thereby exacerbating psoriasis-like inflammation and epidermal proliferation (56).

Metabolic environmental changes are equally critical: keratinocyte cholesterol levels are significantly reduced in psoriasis patients. Experimental work, including patient-derived keratinocyte models, suggests that both cholesterol supplementation and statin treatment can reduce keratinocyte hyperproliferation, although these two interventions act through distinct mechanisms—cholesterol supplementation restores membrane lipid homeostasis and barrier integrity, whereas statins inhibit the mevalonate pathway and thereby reduce downstream inflammatory mediators and protein prenylation—indicating that metabolic stress and genetic susceptibility jointly determine the keratinocyte response threshold to environmental stimuli (57).

## Inflammatory initiation: keratinocyte-mediated innate immune activation

### IL-1 and IL-36 family: core drivers in pustular psoriasis

IL-36, a member of the IL-1 superfamily, occupies a central position in inflammation initiation in psoriasis, particularly generalized pustular psoriasis (GPP). IL-36 comprises three agonist isoforms (IL-36alpha, IL-36beta, IL-36gamma) and one antagonist (IL-36Ra). Agonists bind the receptor, recruit IL-1 receptor accessory protein, and activate NF-kappaB and mitogen-activated protein kinase (MAPK) pathways, while the antagonist maintains homeostasis by blocking this process (16). In GPP patients, loss-of-function variants in IL36RN cause IL-36Ra deficiency, leading to excessive mature IL-36gamma stimulation of epidermal IL-8 and precursor IL-36gamma production, while downregulating cornified envelope proteins such as corneodesmosin, promoting neutrophil infiltration and Kogoj spongiform pustule formation (17, 18). This keratinocyte-driven IL-36 imbalance forms a positive feedback loop leading to uncontrolled inflammation and pustular flares (19). It should be emphasized that IL-36 biology is exceptionally important in GPP and pustular subtypes; evidence derived from GPP should not be automatically generalized to chronic plaque psoriasis, and the disease subtype should be stated whenever evidence is introduced. Clinical studies confirm that the anti-IL-36 receptor biologic spesolimab rapidly downregulates skin IL-36 isoforms and downstream chemokines (CXCL1/6/8) during acute GPP flares, significantly clearing pustules and improving systemic inflammation (20, 21). The IL-1 pathway also participates in pustular psoriasis initiation: NLRP1 inflammasomes activated by endogenous double-stranded RNA mediate IL-1 release via Gasdermin D pores, further inducing keratinocyte IL-36gamma secretion (22); isolated reports of pustular eruptions following vaccination have described clinical improvement with the IL-1 inhibitor anakinra, providing case-level support for IL-1-mediated autoinflammation (58). Notably, IL-36 and IL-23 signaling are closely linked, as IL-23 pathway inhibition reduces keratinocyte IL-36 expression, indicating that innate and adaptive immune signals converge at the initiation stage (10).

### LL-37: dual function as autoantigen and immune adjuvant

Keratinocyte-derived LL-37 plays a dual role as autoantigen and immune adjuvant in psoriasis initiation. As an autoantigen, LL-37-nucleic acid complexes activate plasmacytoid DCs to produce interferon-alpha, promoting myeloid DC maturation and Th1/Th17 differentiation (24). As an adjuvant, LL-37 activates TLR8 on keratinocytes to induce IL-17C production, a process dependent on IL-36gamma upregulation; in psoriatic lesions, LL-37, TLRs, IL-17C, and IL-36gamma show co-expression patterns, and IL-36 receptor blockade inhibits LL-37-induced IL-17C expression (25). Furthermore, LL-37-dsRNA complexes activate RIG-I-like receptor signaling, leading to Gasdermin E cleavage and IL-36gamma release from keratinocytes in a cell death-independent manner, or promote IL-36gamma expression and processing through NLRP1 inflammasome activation (26). These multiple activation mechanisms make LL-37 a key molecule linking environmental triggers to epidermal innate immune responses. Reverse regulatory mechanisms also corroborate the importance of LL-37: Sprouty1 overexpression reduces cathelicidin production through Stat3 inhibition, thereby alleviating imiquimod-induced skin inflammation and DC and gammadeltaT cell infiltration (46). These findings indicate that LL-37 is not merely an effector molecule but an active initiator of the early inflammatory cascade in psoriasis.

### Danger signal release and early dendritic cell recruitment

Keratinocytes actively recruit and activate DCs by releasing danger signals and chemokines, constituting an early key step in inflammation initiation. In the inflammatory microenvironment, keratinocytes secrete IL-36 and IL-23 to directly interact with DCs (7), and through epigenetic modifications such as lncRNA UCA1 interaction with METTL14, activate HIF-1alpha and NF-kappaB pathways to enhance innate immune gene expression and inflammatory factor secretion (34). Mitochondrial dysfunction is also an important source of danger signals: HDAC3 upregulation causes mitochondrial DNA leakage into the cytosol, activating the cGAS-STING pathway in keratinocytes, which synergistically induces NF-kappaB activation and pro-inflammatory factor expression (59, 60). At the intercellular communication level, spatial single-cell work in palmoplantar pustulosis has identified keratinocyte/endothelial chemokine programs involving CXCL1/6/8 and ACKR1 that support neutrophil recruitment and local immune-niche organization (61). Although some studies emphasize the role of the TLR7-MyD88 axis in DC CXCL16 secretion and neutrophil activation (62), keratinocytes can contribute IL-17C, IL-36gamma, and DAMPs that help shape the local microenvironment for DC activation and pathogenic T cell polarization (15, 63). Furthermore, keratinocytes can function as non-professional antigen-presenting cells, directly activating CD4+ T cells through MHC-II molecules loaded with K17 epitopes, further amplifying the inflammatory response initiated by DCs (11). This danger-signal network helps coordinate the local transition from innate to adaptive immune responses (see **Table 2** for a complete overview of key keratinocyte-derived mediators in psoriatic inflammation, stratified by functional category).

**Table 2:** Key keratinocyte-derived mediators in psoriatic inflammation, stratified by functional category.

Mediator	Category	Function in psoriasis	Ref.
IL-36alpha/beta/gamma	Cytokine (IL-1 family)	Core driver of GPP; NF-kappaB/MAPK activation; neutrophil recruitment	(16–21)
IL-1beta/IL-18	Cytokine (inflammasome)	Pyroptosis release; innate immune amplification	(22, 27, 29, 45)
IL-23	Cytokine	Keratinocyte-derived; drives Th17; linked to IL-36	(10, 64)
LL-37 (cathelicidin)	Antimicrobial peptide	Autoantigen + adjuvant; pDC activation; TLR8/IL-17C	(24–26, 46)
S100A8/A9	Antimicrobial peptide/DAMP	TLR-dependent inflammatory signaling (including TLR4-associated pathways); neutrophil-associated inflammation; epigenetic regulation	(48–50)
Beta-defensins (HBD-2/3)	Antimicrobial peptide	Innate defense; copy number variation linked to psoriasis risk	(47)
CXCL1/6/8	Chemokine	Neutrophil recruitment; endothelial ACKR1 interaction (PPP evidence)	(61)
CCL20	Chemokine	CCR6+ cell recruitment; Th17/Tc17 homing	(80, 122)
TNF-alpha	Cytokine	Keratinocyte-derived; amplifies inflammation; therapeutic target	(14, 125)
IL-17C	Cytokine	LL-37/TLR8-induced; autocrine keratinocyte activation	(25)
Extracellular vesicles (EVs)	Intercellular carrier	LRG1-mediated macrophage activation; cfNA/TLR signaling	(14, 68, 104)
Neuropeptides/OXTR	Neuro-immune	Sensory-neuron crosstalk; IL-17RA regulation; itch-associated signaling	(5, 36, 71)
DAMPs/cytosolic DNA (including mtDNA)	Danger signal	cGAS-STING signaling; inflammasome activation; sterile inflammation	(59, 60)
Lactate/metabolites	Metabolic mediator	Histone lactylation; HIF-1alpha-linked metabolic remodeling	(70, 76, 77)

## Inflammatory maintenance and amplification: active regulation of adaptive immunity by keratinocytes

Keratinocytes are not only passive targets in psoriasis pathogenesis but also key hubs that drive inflammation initiation and maintenance through secretion of multiple mediators (7, 8). This active regulatory role manifests in complex bidirectional communication networks formed with adaptive immune cells, establishing self-amplifying cytokine loops such as the IL-23/Th17 axis that sustain local inflammation (5, 64). Keratinocyte-derived IL-23 is closely associated with IL-36 expression, and inhibition of IL-23 signaling reduces keratinocyte IL-36 expression, supporting a contribution of epidermal IL-23/IL-36 signaling to local inflammatory circuits (10). Keratinocytes also release DAMPs through programmed cell death, dynamically interacting with immune cells and cytokines to form complex regulatory networks that maintain chronic inflammation (15). This keratinocyte-driven positive feedback mechanism enables psoriatic lesions to persist long after removal of the initial trigger, underscoring the importance of targeting keratinocytes to break the inflammatory cycle (5, 8).

### Chemokine network: directed recruitment and retention of Th17/Tc17 cells

Keratinocytes precisely regulate the recruitment and *in situ* retention of pathogenic T cells such as Th17 and Tc17 in the epidermis by constructing a specific chemokine microenvironment. In the psoriatic microenvironment, keratinocytes respond to stimuli such as IL-17A by producing not only classical chemokines but also enhancing T cell infiltration through non-classical pathways. For example, IL-17A can induce CXCL16 expression in keratinocytes; in an experimental treatment study, reduced Cxcl16 expression was accompanied by lower Th17 proportions and improved skin inflammation (65). Beyond direct chemokine secretion, keratinocytes indirectly promote T cell activation and homing by processing self-antigens. Transcobalamin 1 (TCN1), a self-antigen secreted by keratinocytes, can be internalized and presented by antigen-presenting cells to CD4+ T cells, promoting Th17 differentiation, and TCN1 expression levels positively correlate with lesional IL-17 signal intensity and keratinocyte proliferation (66). More critically, keratinocytes themselves can function as non-professional antigen-presenting cells: K17-positive keratinocyte subsets express MHC-II molecules loaded with K17-derived epitopes and can directly activate CD4+ T cells in an HLA-restricted manner; adoptive transfer of K17-specific T cells significantly exacerbates psoriasis-like skin inflammation, confirming the central role of direct keratinocyte-T cell interaction in inflammation amplification (11). Compartmentalized dysregulation of calcium signaling also participates: sustained calcium-dependent activation in immune cells promotes Th17 polarization through the Calcineurin-NFAT-RORgammat pathway, while impaired calcium signaling in keratinocytes leads to barrier defects, together constituting a compartment-specific bidirectional calcium dysregulation pathological pattern that further stabilizes the pathogenic effects of Th17 cells in lesions (67).

### Exosome-mediated intercellular communication and immune microenvironment remodeling

Keratinocyte-derived EVs function as novel intercellular communication carriers that play a non-negligible role in remodeling the psoriatic immune microenvironment and amplifying inflammatory cascades. In an imiquimod-induced psoriasis-like dermatitis model, keratinocyte-released EVs were shown to recruit macrophages and promote skin lesion progression; *in vitro* experiments demonstrate that EVs from keratinocytes stimulated by inflammatory factors significantly promote monocyte expression of IL-1beta and tumor necrosis factor-alpha (TNF-alpha), with LRG1-enriched EVs regulating macrophage activation through a TGFbeta receptor 1-dependent pathway, revealing a novel pro-inflammatory interaction mechanism between keratinocytes and macrophages mediated by EVs (14). Beyond protein components, nucleic acids carried by EVs can act as immune regulators. In a non-psoriatic UVB-damage model, keratinocyte-derived exosomal cell-free nucleic acids (cfNAs) activated endosomal TLR3/TLR9 and downstream NF-kappaB signaling, while cfNA capture reduced sterile cutaneous inflammation (68). This finding supports the broader plausibility of EV nucleic acids as DAMP carriers, but it should not be interpreted as psoriasis-specific evidence. Collectively, EV-mediated communication networks greatly expand the dimension of keratinocyte regulation of the immune microenvironment, enabling remote remodeling of immune cell function without direct contact.

### Neuropeptide-keratinocyte axis in the itch-scratch cycle

Keratinocytes are not only structural components of the physical barrier but also key nodes of neuro-immune interaction, participating in itch-associated signaling through neuropeptides and inflammatory mediators (5). In chronic psoriatic inflammation, keratinocytes form close functional coupling with sensory nerve endings. Innervated *in vitro* psoriasis models show that sensory neuron-derived calcitonin gene-related peptide (CGRP) can increase keratinocyte proliferation (36). Metabolic remodeling may intersect with this neuroimmune circuitry: psoriasis studies demonstrate HIF1alpha-linked glycolytic remodeling and keratinocyte H4K12 lactylation as amplifiers of IL-17-dependent proliferation (69, 70), although direct evidence that lesional lactate itself sensitizes sensory neurons in psoriasis remains limited. Furthermore, the oxytocin receptor (OXTR) has been identified as a regulator of IL-17 receptor A surface expression, and topical OXTR antagonism suppresses imiquimod-induced dermatitis in mice with efficacy comparable to systemic anti-IL17RA treatment (71). Together, these findings support bidirectional neuroimmune crosstalk involving keratinocytes, while the specific contribution of this axis to clinical itch requires further validation.

## Metabolic reprogramming and barrier disruption: keratinocyte homeostatic imbalance

Psoriasis has been redefined as a metabolically regulated inflammatory skin disease, with the core feature that keratinocytes are not merely passive targets of immune responses but active drivers of metabolic dysregulation (2, 72). Studies confirm that keratinocytes in psoriatic lesions exhibit significant metabolic reprogramming involving comprehensive abnormalities in glycolysis, oxidative phosphorylation, lipid metabolism, and amino acid metabolism, establishing a reciprocal cycle between metabolic remodeling and immune dysfunction (72). Keratinocytes amplify immune signals through abnormal metabolic products, while cytokines in the immune microenvironment further exacerbate metabolic dysregulation, forming a difficult-to-break pathological closed loop (72, 73). Furthermore, metabolic stress and mitochondrial dysfunction directly impair epidermal barrier integrity, transforming keratinocytes from simple physical barriers into sustained sources of pro-inflammatory signals (74, 75). In-depth analysis of this metabolic-barrier-immune axis imbalance is critical for understanding the chronic progression of psoriasis and developing novel therapies targeting metabolic checkpoints (72, 73).

### Carbohydrate and lipid metabolic abnormalities driving keratinocyte hyperproliferation and differentiation defects

Psoriatic keratinocytes exhibit Warburg-like metabolic features that support rapid proliferation and biosynthesis (76). In broader epidermal biology, constitutive HIF-1alpha activity can sustain proliferation and barrier programs (77), while psoriasis-specific studies show that HIF-1alpha stability is increased through SIRT7-mediated deacetylation, upregulating glycolysis-related gene expression (41). K17 interacts with alpha-enolase (ENO1) and maintains its serine phosphorylation, promoting lactate dehydrogenase A (LDHA) transcription and directly enhancing glycolytic flux (32). Additionally, CD147, an interacting protein of glucose transporter 1 (Glut1), promotes glucose uptake and inhibits oxidative phosphorylation; its deletion restores carnitine and alpha-ketoglutarate levels, alleviating psoriasis-like inflammation (78). Notably, basal cells preferentially take up glucose through metabolic competition, upregulating COX7B-driven oxidative phosphorylation and producing reactive oxygen species (ROS) to promote proliferation, whereas suprabasal cells exhibit disulfide stress and immature differentiation due to glucose deprivation, revealing the role of metabolic heterogeneity in tissue homeostasis disruption (79). In lipid metabolism, long-chain fatty acid levels are significantly elevated in psoriatic lesions, entering keratinocytes through upregulated CD36 and causing lipid accumulation and mitochondrial dysfunction (80); patient metabolomic profiling also indicates impaired beta-oxidation and increased stearoyl-CoA desaturase (SCD) activity (81). Glutamine metabolism is also reprogrammed: as a dual nitrogen-carbon donor, its catabolic product alpha-ketoglutarate not only provides ATP for proliferation but also triggers inflammation through mTOR, NF-kappaB, and STAT3 pathways (73). These metabolic changes collectively contribute to keratinocyte hyperproliferation and terminal differentiation defects in psoriatic plaques.

### Mitochondrial dysfunction and ROS-mediated feedback enhancement of inflammatory signals

Mitochondrial dysfunction is a component of inflammatory amplification in psoriatic keratinocytes, manifesting as mitochondrial DNA (mtDNA) damage, metabolic pathway alterations, and excessive ROS production (75, 82). In a psoriasis-like mouse model, a mitochondria-targeted probe for vicinal dithiol-containing proteins demonstrated marked redox imbalance associated with disease activity (83). ROS are not merely byproducts of metabolic damage but signaling mediators: CD36-mediated long-chain fatty acid accumulation induces mitochondrial ROS generation, activating NF-kappaB signaling and upregulating CXCL2 and CCL20 expression (80); high levels of glucose metabolism-derived ROS in basal cells, although partially buffered by the glutathione system, continuously drive proliferation signals (79). However, the role of ROS is complex: p67phox/NOX2 is upregulated in psoriatic epidermis, and its knockdown paradoxically leads to AMPK inactivation and HIF-1alpha stabilization, exacerbating metabolic lactylation and inflammation, suggesting that specific ROS signals may have a protective role in maintaining metabolic homeostasis (84). Ferroptosis, a metabolic form of cell death driven by lipid peroxidation and iron homeostasis dysregulation, has been implicated in psoriatic lesions as a link between metabolic stress and inflammatory signaling (85). Preclinical psoriasis studies support redox-targeted intervention as a therapeutic concept (86), whereas evidence for PGC-1alpha/NAD+ support and related mitochondrial-restorative strategies derives largely from general epidermal or skin-aging models (87, 88); therefore, psoriasis-specific translational evidence remains limited (82).

### Epidermal barrier protein defects and microbiome dysregulation

Epidermal barrier dysfunction is not merely a clinical consequence of psoriasis but may contribute to inflammation initiation and maintenance (74). Evidence from non-psoriatic barrier models shows that Gsdma1/a3 deletion can impair tight-junction formation and stratum-corneum maturation with associated mitochondrial dysfunction (89); this supports a general role for gasdermin A in barrier homeostasis but is not psoriasis-specific evidence. Altered serpin expression has also been reported in psoriasis, including SERPINA12 dysregulation, although the causal contribution of individual serpin-family members to psoriatic barrier failure remains incompletely defined (90). Lipid metabolic abnormalities further disrupt the physical integrity of the barrier: ceramide metabolism genes such as UGCG are regulated by the aryl hydrocarbon receptor (AHR), and AHR activation can promote epidermal glucosylceramide synthesis (91). Conversely, CD36-mediated lipid accumulation and mitochondrial damage are directly associated with psoriasis-like inflammation (80). Barrier disruption and microbiome dysregulation may form a bidirectional cycle: an impaired barrier facilitates microbial-product access to innate immune receptors, while keratinocyte antimicrobial peptides and danger signals alter the local microenvironment (2, 8). Microdialysis metabolomics analysis shows that lesional areas exhibit elevated nucleotide/pyrimidine metabolites and polyamines, with decreased histidine metabolism, and these metabolic features are associated with disease severity (92). Single-cell and spatial studies also suggest that clinically non-lesional skin can harbor subclinical molecular abnormalities (69). Thus, restoring keratinocyte metabolic homeostasis and barrier integrity is a plausible complementary strategy for breaking barrier-immune feedback.

## Diagnostic biomarker exploration: assessment systems based on keratinocyte activity

As understanding of psoriasis pathogenesis deepens, keratinocytes have been redefined from simple inflammatory targets to key drivers of disease initiation and maintenance. This paradigm shift urgently requires novel diagnostic assessment systems that can precisely reflect keratinocyte activation status, distinguish disease subtypes, and monitor therapeutic response. Existing clinical scores such as the Psoriasis Area and Severity Index (PASI), although widely used for clinical severity assessment, cannot capture intrinsic epidermal molecular pathological changes or microscopic alterations in pre-psoriatic states (93, 94). Therefore, integrating transcriptomic, proteomic, and metabolomic data to identify biomarkers that specifically reflect keratinocyte immune activity and metabolic reprogramming has important translational value for precise phenotyping, therapeutic response prediction, and long-term management of psoriasis (8, 95).

### Candidate biomarkers reflecting keratinocyte activation status

Abnormal keratinocyte activation involves multiple dimensions including proliferation, differentiation defects, metabolic reprogramming, and innate immune signal amplification, and related molecules have emerged as promising candidate biomarkers. It is essential to distinguish between exploratory candidate biomarkers identified in mechanistic or discovery studies and clinically validated biomarkers; most of the molecules described below remain at the discovery or proof-of-concept stage and require independent replication, prospective validation, predefined thresholds, calibration, discrimination metrics, clinical utility studies, and comparison with standard assessments before they can be described as diagnostic or predictive biomarkers. In proliferation and differentiation regulation, PA2G4 is highly expressed in basal proliferative keratinocytes of psoriatic lesions, with levels positively correlated with epidermal thickening, neutrophil infiltration, and upregulation of psoriasis-related genes such as MKI67 and IL20; PA2G4 knockout reverses the proliferative phenotype and promotes differentiation, suggesting its potential as a marker of keratinocyte hyperproliferative activity (96). YAP1, a Hippo pathway effector, is significantly upregulated in psoriatic skin and models, directly driving keratinocyte proliferation and inflammation, and its antagonists effectively alleviate lesions, supporting YAP1 as an exploratory candidate marker of keratinocyte pathological hyperplasia (97, 98). Additionally, SLC35E1 is highly expressed in psoriatic patient keratinocytes, regulating zinc homeostasis to affect cell proliferation, with expression associated with disease phenotype severity, supporting its consideration as an exploratory mechanistic marker (99).

In metabolic and inflammatory reprogramming, epidermal fatty acid binding protein FABP5 is significantly upregulated in psoriatic epidermis; keratinocyte-specific FABP5 deficiency reduces neutrophil recruitment, and the FABP5-VCP complex promotes NF-kappaB-dependent chemokine signaling (100). These findings support FABP5 as a mechanistically grounded candidate biomarker and therapeutic target rather than a clinically validated biomarker. Elevated K17 expression is associated with glycolytic pathway activation, and its interaction with ENO1 promotes LDHA transcription and keratinocyte proliferation, making the ENO1-K17 axis a candidate marker of metabolic activation (32). In innate immune and inflammatory signaling, OASL is significantly upregulated in psoriatic lesions and promotes hyperproliferation and metabolic-inflammatory crosstalk through MAPK signaling, supporting its evaluation as an exploratory marker (101). HDAC3 upregulation promotes oxidative stress and mitochondrial DNA leakage, activating the cGAS-STING pathway, and may serve as a mechanistic indicator of epigenetically regulated keratinocyte innate immune activation (59). Programmed cell death-related molecules such as Granzyme K and necroptosis pathway components also show potential as exploratory markers of keratinocyte damage and inflammatory feedback (15, 102, 103). In circulating biomarker studies, plasma EV miR-625-3p derived from stimulated keratinocytes has been associated with psoriasis severity, and APOA1, DEFB103A/B, and DSG3 have been reported as candidate circulating biomarkers (104, 105). Computational lactylation-signature analyses have also proposed additional exploratory biomarker candidates, but these remain discovery-stage findings (106). Independent prospective validation is still required before clinical deployment.

### Molecular signatures distinguishing psoriasis subtypes

Clinical phenotypic heterogeneity in psoriasis requires a precise phenotyping system based on molecular signatures to guide individualized treatment. IL-36 pathway activity is particularly prominent in generalized pustular psoriasis (GPP), whereas plaque psoriasis shows a broader immune-epithelial network (16, 18). At the protein-marker level, IL-36gamma and the elastase-specific protease inhibitor elafin have been reported as epidermal markers that can help distinguish psoriasis from atopic dermatitis; elafin showed higher diagnostic performance than IL-36gamma in the cited proteomic study (107). This evidence does not by itself validate IL-36gamma as a discriminator between pustular and plaque psoriasis. In transcriptomic stratification, unsupervised clustering based on gene microarray data from 250 patient lesions identified three molecular subtypes: subtype A (immune activation) enriched for immune cells and pro-inflammatory pathways; subtype C (stromal proliferation) enriched for stromal cells and tissue proliferation pathways; and subtype B showing moderate activation. This phenotyping not only distinguishes disease biology but was associated with differential therapeutic response in the study cohort: subtypes A/B responded better to methotrexate and IL-12/23 inhibitors, whereas subtype C showed greater response to TNF-alpha and IL-17A receptor inhibition (108). Additionally, phospholipases PLA2G2F, PLA2G4D, and PLA2G4E are enriched in plaque psoriasis epidermis and influence eicosanoid metabolism, supporting their investigation as lipid-metabolic markers (109). At the differential-diagnosis level, a real-time PCR-based molecular classifier using formalin-fixed paraffin-embedded tissue, microbiopsies, or tape strips can distinguish psoriasis from eczema with high reported sensitivity and specificity (110). Differential galectin-expression patterns have also been proposed as potentially informative in psoriasis differential diagnosis (111).

### Minimally invasive detection technologies for monitoring epidermal immune activity

Given the chronic relapsing nature of psoriasis and the invasiveness of epidermal sampling, development of high-sensitivity, high-resolution minimally invasive detection technologies is critical for dynamic monitoring of keratinocyte immune activity. Tape stripping, a minimally invasive sampling method combined with high-throughput RNA sequencing, has been shown to capture the upper epidermal transcriptome with sufficient resolution to effectively assess molecular components of the psoriasis positive feedback immune amplification pathway. Importantly, this technique can detect pro-inflammatory alterations in clinically normal non-lesional skin that full-thickness biopsies fail to capture, revealing the molecular basis of pre-psoriatic states and providing a new tool for early disease warning and relapse monitoring (94). Molecular classifiers based on tape-stripped or micro-biopsy samples have been successfully applied to differential diagnosis of psoriasis and atopic dermatitis, validating the reliability of minimally invasive samples in molecular diagnosis (110). Skin microdialysis, by collecting interstitial fluid from lesional and non-lesional areas combined with untargeted proteomics and metabolomics analysis, reveals significantly elevated SERPINB3/4, FABP5, and specific nucleotide and polyamine metabolites in lesional areas, and these molecular features normalize with systemic treatment, confirming its potential as a real-time monitoring tool for therapeutic response (92). In sensing technology, a surface-enhanced Raman scattering (SERS) skin patch based on electrospun flexible silver triangular nanosheets enables *in situ*, rapid, and highly sensitive detection of human beta-defensin 2 (HBD-2) (detection limit 59.57 fg/mL), opening a new path for point-of-care monitoring of keratinocyte antimicrobial peptide activity (112). In liquid biopsy, plasma EV miRNAs such as miR-625-3p, as signal carriers secreted by keratinocytes, have levels synchronized with disease severity and histopathological changes, serving as circulating surrogate markers for systemic monitoring of epidermal immune activity (104). The integrated application of these minimally invasive technologies is driving psoriasis diagnosis and treatment from static histopathological assessment toward dynamic molecular functional monitoring.

## Therapeutic innovation: dual intervention pathways targeting keratinocytes

As understanding of psoriasis pathogenesis deepens, keratinocytes have been transformed from simple inflammatory targets into core hubs for therapeutic intervention. Current treatment strategies are undergoing a paradigm shift from single T cell suppression to immune-barrier dual regulation, aiming to achieve precise interruption of the inflammatory cycle and long-term homeostatic maintenance by blocking keratinocyte-mediated innate immune initiation signals, repairing epidermal barrier function, and correcting metabolic reprogramming abnormalities (5, 7). This dual intervention pathway encompasses not only the development of novel biologics targeting key pro-inflammatory axes such as IL-36/IL-1 but also topical agents and physical therapies that can directly regulate keratinocyte differentiation and metabolic status, providing new theoretical basis and clinical options for refractory psoriasis and specific subtypes (8, 113). It is important to establish a clear evidence hierarchy: approved therapies, human observational evidence, isolated clinical cases, mouse experiments, and cell culture studies represent different levels of evidence and should not be presented as equivalent.

### Targeting IL-36/IL-1 pathways: biologics in refractory psoriasis

The central pathogenic role of the IL-36 cytokine family as regulators of epithelial barrier inflammation has been confirmed in GPP and supported by preclinical evidence in plaque-psoriasis models (16, 114). Spesolimab, a first-in-class humanized monoclonal antibody that specifically binds the IL-36 receptor (IL-36R) and blocks downstream signaling, received FDA approval in 2022 for the treatment of GPP flares in adults; clinical studies demonstrate rapid improvement in pustulation and systemic inflammatory signatures (20, 21, 115). In contrast, plaque-psoriasis evidence for IL-36R blockade remains preclinical: anti-IL-36R treatment suppresses IL-17A-driven and imiquimod-induced psoriasis-like inflammation in mice, including keratinocyte pro-inflammatory gene expression and immune-cell activation (116). Small-molecule antagonist development is also advancing. IRA10L competitively binds IL-36R and inhibits downstream inflammatory mediators in experimental systems, establishing proof of concept for small-molecule IL-36R antagonism, but psoriasis efficacy has not yet been established (117). Notably, the IL-36 and IL-17 pathways are closely interconnected: transcriptomic analysis of the IXORA-R trial shows that IL-17A inhibition produces early downregulation of IL-36-response genes in lesions, and these changes are associated with rapid clinical response (118). For GPP patients with IL36RN loss-of-function variants, excessive IL-36 signaling contributes to epidermal dysfunction and pustule formation, illustrating the rationale for pathway-directed treatment (17).

### Topical agents for barrier repair and differentiation regulation

Given that keratinocyte differentiation defects and barrier disruption contribute to persistent psoriatic inflammation, topical agents with both anti-inflammatory and barrier-restorative effects are of interest (113, 119). Established topical therapies including vitamin D analogs and topical corticosteroids act at least in part through keratinocyte differentiation and inflammatory signaling. More recently, psoriasis-specific preclinical work has explored compounds that directly restore keratinocyte homeostasis. Entadamide A-beta-D-glucopyranoside (EA) inhibits keratinocyte hyperproliferation and restores differentiation markers in psoriasis models, with proposed mechanisms involving IL-36R/P2X7R signaling and NLRP3 inflammasome inhibition (120). Targeting the protease meprin alpha, which is dysregulated in psoriatic lesions and linked to barrier-protein processing, has also been proposed as a barrier-directed strategy (119). Small extracellular vesicles from adipose-derived mesenchymal stem cells can promote epidermal lipid synthesis and differentiation through sphingosine-1-phosphate signaling in general barrier models (121); however, this evidence is not psoriasis-specific and should be viewed as hypothesis-generating for psoriasis. Overall, barrier-directed therapy remains a plausible complement to immunomodulation, but candidate approaches require disease-specific validation before clinical translation.

### Physical therapy and metabolic modulators correcting keratinocyte immune function

Keratinocyte immune function is tightly coupled to metabolic status, making metabolic modulation a promising but largely preclinical therapeutic direction (5). AMPK activators AICAR and A769662 inhibit IL-36-induced IL-8 and CCL20 production in human keratinocytes and reduce IL-36-driven skin inflammation in mice (122). Serine metabolism has also emerged as a regulatory node: the SHMT1/2 inhibitor SHIN1 reduces IL-22-driven keratinocyte proliferation and IFN-gamma-induced inflammatory responses in psoriasis models (123). In a separate psoriasis study, S-adenosylmethionine (SAM) supplementation mitigated inflammation by restoring methyl-donor availability and DNA methylation linked to NF-kappaB/IL-6 signaling (124). Concentrated growth factor (CGF) has been investigated using clinical observations, animal models, and *in vitro* keratinocyte experiments, but the evidence remains preliminary and requires controlled validation (125). These findings indicate that metabolic interventions can modulate keratinocyte inflammatory phenotypes; however, entadamide, AMPK modulators, SHIN1, SAM, CGF, and extracellular-vesicle approaches remain preclinical or early-stage and should not be presented as established psoriasis therapies.

## Discussion

### Conceptual integration: keratinocytes within epithelial-immune reciprocity

The evidence reviewed here supports a conceptual reframing of psoriasis pathogenesis: rather than a disease driven primarily by adaptive immune cells attacking a passive epidermal target, psoriasis is best understood as a disorder of bidirectional epithelial-immune reciprocity, in which keratinocytes are active and necessary participants. This reframing does not diminish the central importance of T cells, DCs, and the IL-23/Th17 axis; rather, it positions keratinocytes as active partners that initiate, amplify, and sustain inflammatory circuits through cytokine secretion, antigen presentation, metabolic reprogramming, barrier disruption, and intercellular communication via EVs and neuropeptides. The historical pendulum—from keratinocyte-centric disease to immune-centric disease and now toward an integrated model—reflects the maturation of multi-omics and functional genomic approaches that can resolve cell-type-specific contributions.

A comparative analysis across inflammatory skin diseases underscores the context-dependence of keratinocyte immune activity. In atopic dermatitis, keratinocyte barrier defects and type 2 immune responses predominate, whereas in psoriasis, the IL-23/Th17/IL-17 axis and IL-36-driven autoinflammation are more prominent; evidence derived from atopic dermatitis or radiodermatitis should not be treated as psoriasis-specific. Within the psoriasis spectrum, GPP is driven predominantly by IL-36 pathway dysregulation (often with IL36RN mutations), palmoplantar pustulosis has distinct neutrophilic and barrier features, and chronic plaque psoriasis involves a broader immune-epithelial network. Generalizing IL-36 evidence from GPP to plaque psoriasis without qualification risks overstating its role in the latter. Disease endotype should be stated whenever evidence is introduced.

### Controversies and conceptual challenges

Several controversies remain. First, the causal direction of keratinocyte-immune interactions is not fully resolved: while keratinocyte-specific manipulations in mice (e.g., A20 deletion) can initiate psoriasis-like inflammation, it remains unclear whether keratinocyte-intrinsic defects are sufficient to initiate human disease in the absence of immune priming, or whether they primarily lower the threshold for immune-driven inflammation. Second, the relative contributions of different keratinocyte subsets—basal proliferative cells, suprabasal differentiating cells, and recently identified inflammatory keratinocyte subpopulations expressing IL-36G and antiviral proteins—remain to be functionally dissected. Third, the therapeutic implications of targeting keratinocytes versus immune cells require careful weighing: biologics targeting IL-17 and IL-23 are highly effective but do not fully restore barrier function, whereas keratinocyte-directed approaches may offer complementary benefit but remain largely preclinical.

The genetic evidence base requires careful stratification. Common GWAS susceptibility loci, rare monogenic disorders that phenocopy psoriasis, differential gene expression in lesional tissue, keratinocyte-specific functional manipulations in animal models, causal-variant colocalization, and bioinformatically prioritized genes represent fundamentally different evidence classes. Conflating them obscures the distinction between population-level risk and cell-intrinsic mechanism. For example, ADAR1 loss-of-function causing an interferon-dependent psoriasis-like phenotype is biologically interesting but conceptually distinct from common polygenic susceptibility; keratinocyte-specific A20 deletion in mice is functional mechanistic evidence, not human population-genetic evidence. Future genetic studies should explicitly classify evidence type and prioritize functional validation of candidate genes in human keratinocyte systems (see **Table 3** for an evidence-stratified overview of therapeutic strategies targeting keratinocytes)

**Table 3:** Therapeutic strategies targeting keratinocytes, stratified by evidence level.

Therapy/target	Evidence level	Keratinocyte-relevant mechanism	Ref.
Spesolimab (anti-IL-36R)	Approved (GPP)	Blocks IL-36 signaling; inhibits keratinocyte pro-inflammatory genes	(20, 21, 115, 116)
Vitamin D analogs	Approved (topical)	Regulates keratinocyte proliferation/differentiation via VDR	Standard of care
Topical corticosteroids	Approved (topical)	Suppresses keratinocyte pro-inflammatory gene expression	Standard of care
Methotrexate	Approved (systemic)	Antiproliferative; affects keratinocyte and immune cell metabolism	Standard of care
Tapinarof (AHR agonist)	Approved (topical)	Upregulates ceramide synthesis; barrier repair; anti-inflammatory	(91, 126)
TYK2 inhibitors (deucravacitinib)	Approved (systemic)	Selective TYK2 inhibition; suppresses cytokine signaling relevant to psoriasis; systemic deucravacitinib clinically validated.	(38, 127)
IL-17/IL-23 biologics	Approved (systemic)	Indirectly modulates keratinocyte via cytokine milieu; does not fully repair barrier	(37, 118)
Anakinra (IL-1Ra)	Approved (other indications)	IL-1 blockade; effective in pustular psoriasis cases	(58)
IRA10L (IL-36R antagonist)	Preclinical/non-psoriasis efficacy unestablished	Small-molecule IL-36R antagonist; proof-of-concept only	(117)
Entadamide A (EA)	Preclinical	IL-36R/P2X7R binding; NLRP3 inhibition; differentiation restoration	(120)
AMPK activators (AICAR/A769662)	Preclinical	IkappaBzeta degradation; inhibits IL-8/CCL20 in keratinocytes	(122)
SHIN1 (SHMT1/2 inhibitor)	Preclinical	Serine metabolism blockade; reduces proliferation and IFN-gamma response	(123)
SAM (methyl donor)	Preclinical	DNA methylation restoration; IL-6 suppression	(124)
CGF (concentrated growth factor)	Preclinical/early clinical	CD34+ cell-mediated; reduces keratinocyte TNF/IFN/IL-23 secretion	(125)
ASC-sEVs (exosomes)	Preclinical/non-psoriasis barrier model	Ceramide synthase; S1P pathway; barrier lipid synthesis	(121)
Meprin alpha inhibitors	Preclinical	Restores dermokine processing; improves barrier integrity	(119)
Mitochondrial-targeted antioxidants	Preclinical/cross-context evidence	Redox/mitochondrial restoration; psoriasis-specific validation limited	(82, 86–88)

### Research gaps and future directions

Critical research gaps remain. First, the lack of validated clinical biomarkers is a major limitation: most molecules described in this review are exploratory candidates identified in mechanistic or discovery studies, and none have undergone the prospective validation, threshold calibration, discrimination assessment, and clinical utility studies required for clinical deployment. Second, animal models have significant limitations in recapitulating human keratinocyte-immune interactions, including the absence of certain human keratinocyte subpopulations and the difficulty of modeling chronic relapsing disease; development of humanized models and organotypic skin cultures with integrated immune components is urgently needed. Third, the spatial and temporal dynamics of keratinocyte activation—particularly the transition from pre-lesional to lesional states and the mechanisms of relapse after treatment—remain poorly understood.

Future research should prioritize several directions: (i) high-resolution spatial multi-omics to map keratinocyte subpopulations and their interactions with immune cells across disease stages; (ii) functional validation of candidate biomarkers in prospective cohorts with predefined thresholds and clinical utility endpoints; (iii) development of keratinocyte-directed therapies with clear evidence hierarchies, moving from preclinical concepts to rigorous clinical trials; (iv) integration of barrier repair with immune suppression in combination regimens; and (v) explicit disease-endotype stratification in all mechanistic and therapeutic studies. The transition from single-target cytokine blockade to multimodal intervention addressing both immune dysregulation and keratinocyte intrinsic defects represents a promising path toward deeper and more durable disease control in psoriasis.

## Glossary

AHR: aryl hydrocarbon receptor
AIM2: absent in melanoma 2
AMPK: AMP-activated protein kinase
CCL: chemokine (C-C motif) ligand
CD: cluster of differentiation
CGF: concentrated growth factor
CXCL: chemokine (C-X-C motif) ligand
DC: dendritic cell
DAMP: damage-associated molecular pattern
DEFB: defensin beta
EDC: epidermal differentiation complex
ENO1: alpha-enolase
ERAP: endoplasmic reticulum aminopeptidase
EV: extracellular vesicle
FABP5: fatty acid binding protein 5
FXR: farnesoid X receptor
GPP: generalized pustular psoriasis
GSDMD/E: gasdermin D/E
GWAS: genome-wide association study
HBD: human beta-defensin
HIF-1alpha: hypoxia-inducible factor 1-alpha
HLA: human leukocyte antigen
ICOS: inducible T-cell co-stimulator
IL: interleukin
IL-36R: IL-36 receptor
K17: keratin 17
LCE: late cornified envelope
LDHA: lactate dehydrogenase A
LRG1: leucine-rich alpha-2-glycoprotein 1
MAPK: mitogen-activated protein kinase
MHC-II: major histocompatibility complex class II
m6A: N6-methyladenosine
mtDNA: mitochondrial DNA
NF-kappaB: nuclear factor kappa B
NK: natural killer
NLRP: NOD-like receptor protein
OXTR: oxytocin receptor
PASI: Psoriasis Area and Severity Index
PKM2: pyruvate kinase M2
RIG-I: retinoic acid-inducible gene I
RIPK3: receptor-interacting protein kinase 3
ROS: reactive oxygen species
SAM: S-adenosylmethionine
SCD: stearoyl-CoA desaturase
SERS: surface-enhanced Raman scattering
SHMT: serine hydroxymethyltransferase
S1PR2: sphingosine-1-phosphate receptor 2
STAT: signal transducer and activator of transcription
TCN1: transcobalamin 1
TLR: Toll-like receptor
TNF-alpha: tumor necrosis factor-alpha
TYK2: tyrosine kinase 2
UCA1: urothelial cancer associated 1
VDR: vitamin D receptor
